# A Water-Soluble Hydrogen Sulfide Donor Suppresses the Growth of Hepatocellular Carcinoma via Inhibiting the AKT/GSK-3*β*/*β*-Catenin and TGF-*β*/Smad2/3 Signaling Pathways

**DOI:** 10.1155/2023/8456852

**Published:** 2023-03-07

**Authors:** Shao-Feng Duan, Meng-Meng Zhang, Qian Dong, Bo Yang, Wei Liu, Xin Zhang, Hai-Lan Yu, Shi-Hui Zhang, Nazeer Hussain Khan, Dong-Dong Wu, Xiao-Ju Zhang, Juan Cen

**Affiliations:** ^1^School of Pharmacy, Henan University, Kaifeng, Henan 475004, China; ^2^Henan International Joint Laboratory for Chinese Medicine Efficacy, Henan University, Kaifeng, Henan 475004, China; ^3^Henan International Joint Laboratory for Nuclear Protein Regulation, School of Basic Medical Sciences, Henan University, Kaifeng, Henan 475004, China; ^4^Department of Respiratory and Critical Care Medicine, Henan Provincial People's Hospital, Zhengzhou University People's Hospital, Zhengzhou, Henan 450003, China; ^5^Key Laboratory of Natural Medicine and Immune Engineering, School of Pharmacy, Henan University, Kaifeng, Henan 475001, China

## Abstract

Hepatocellular carcinoma (HCC) is a disease with high morbidity, high mortality, and low cure rate. Hyaluronic acid (HA) is widely adopted in tissue engineering and drug delivery. 5-(4-Hydroxyphenyl)-3H-1, 2-dithiol-3-thione (ADT-OH) is one of commonly used H_2_S donors. In our previous study, HA-ADT was designed and synthesized via coupling of HA and ADT-OH. In this study, compared with sodium hydrosulfide (NaHS, a fast H_2_S-releasing donor) and morpholin-4-ium (4-methoxyphenyl)-morpholin-4-ylsulfanylidenesulfido-*λ*5-phosphane (GYY4137, a slow H_2_S-releasing donor), HA-ADT showed stronger inhibitory effect on the proliferation, migration, invasion, and cell cycle of human HCC cells. HA-ADT promoted apoptosis by suppressing the expressions of phospho (p)-protein kinase B (PKB/AKT), p‐glycogen synthase kinase‐3*β* (GSK-3*β*), p‐*β*‐catenin, and also inhibited autophagy via the downregulation of the protein levels of p‐Smad2, p‐Smad3, and transforming growth factor‐*β* (TGF‐*β*) in human HCC cells. Moreover, HA-ADT inhibited HCC xenograft tumor growth more effectively than both NaHS and GYY4137. Therefore, HA-ADT can suppress the growth of HCC cells by blocking the AKT/GSK-3*β*/*β*-catenin and TGF‐*β*/Smad2/3 signaling pathways. HA-ADT and its derivatives may be developed as promising antitumor drugs.

## 1. Introduction

Liver cancer is one of the leading causes of cancer death worldwide [1–4]. It is a heterogeneous, invasive, and drug-resistant malignant disease with poor prognosis [5–7], which is also the most common malignant tumor in the digestive system, with high mortality and low survival rate [3, 8]. Infection with hepatitis viruses and dietary exposure to aflatoxin are the main causes of liver cancer [9]. Hepatocellular carcinoma (HCC) is a highly invasive and most common liver cancer [10–12]. Many factors can contribute to the development of liver cancer, including hepatitis B virus, hepatitis C virus, cirrhosis, alcoholism, obesity, poor diet and inactivity, and mycotoxins [13–18]. Recent studies have shown undifferentiated liver cancer stem cells to be the main cause for the occurrence, metastasis, recurrence, and chemotherapy resistance of liver cancer [11, 19]. Liver cancer patients often die due to difficulties in early diagnosis, missed opportunity for surgical resection, and delayed treatment [3, 6]. Further research is urgent to advance the prevention, diagnosis, and treatment of liver cancer [13, 20].

Hydrogen sulfide (H_2_S) is a water-soluble and colorless gas with the rotten egg odor [21, 22]. The physiological function of H_2_S has been firstly demonstrated in the mammalian brain [23]. H_2_S has been identified as a gaseous signaling molecule, and together with carbon monoxide (CO) and nitric oxide (NO), forms a bioactive gas transmitter group [24–28]. H_2_S plays an important role in signal transduction in a variety of physiological and pathological processes [29–35]. Cystathionine *γ*-lyase (CSE), cystathionine *β*-synthetase (CBS), and 3-mercaptopyruvate sulfurtransferase (3-MST) are three main enzymes involved in endogenous H_2_S biosynthesis under physiological conditions [21, 32, 36, 37]. H_2_S breakdown is accomplished by a mitochondrial pathway that couples H_2_S oxidation into sulfate and thiosulfate to adenosine triphosphate synthesis [32]. In addition to the absorption of H_2_S through diffusion, a small amount of endogenous H_2_S can also be produced by the dietary L-homocysteine through the sulfur transfer pathway. This process is mainly completed by two enzymes, CBS and CSE, which depend on pyridoxal-5′-phosphate. CBS and CSE use cystathionine to convert homocysteine into cysteine, and H_2_S is a byproduct. In addition, 3-MST can also generate H_2_S. It uses mercaptopyruvate to form persulphide intermediately through *α*-ketoglutarate and cysteine transaminase, and then release H_2_S and pyruvate through reduction reaction [26, 29, 36, 38].

Hyaluronic acid (HA) is a glycosaminoglycan widely existing in human body. HA, a natural polysaccharide, is composed of N-acetylglucosamine and glucuronic acid, which are alternately linked by *β*-1, 3 and *β*-1, 4 glycoside bonds [39, 40]. Because HA and its derivatives have the characteristics of high viscoelasticity, plasticity, nonimmunogenicity, good biocompatibility, degradation, and binding with specific receptors on the cell surface, they are often used as slow-release carriers of drugs or as active target ligands of modified nano carriers to achieve the thickening, slow-release, transdermal absorption of drugs and improve the targeting and bioavailability of drugs [39–41]. HA and drug conjugates have been shown to have the dual advantages of tumor site aggregation and receptor-mediated endocytosis [40]. 5-(4-hydroxyphenyl)-3H-1, 2-dithiol-3-thione (ADT-OH) is the most widely used agent in the synthesis of slow H_2_S-releasing donors. ADT is a methyl derivative of ADT-OH, which can be metabolized by mitochondrial enzymes to produce H_2_S [42, 43].

A number of studies have shown that the protein kinase B (PKB/AKT)/glycogen synthase kinase‐3*β* (GSK-3*β*)/*β*-catenin pathway plays an important role in the progression of liver cancer [44–46]. Furthermore, the transforming growth factor‐*β* (TGF‐*β*)/Smad2/3 pathway is involved in the proliferation, migration, invasion, and epithelial-mesenchymal transition (EMT) of liver cancer [47–49]. Diallyl trisulfide, an H_2_S donor, regulates cell invasion and apoptosis via the phosphatidylinositol 3-kinase/AKT/GSK-3*β* signaling pathway in human osteosarcoma U2OS cells [50]. Another study indicates that H_2_S can attenuate paraquat-induced EMT of human alveolar epithelial cells by regulating the TGF-*β*1/Smad2/3 pathway [51]. Our previous study has demonstrated that H_2_S plays a double-edged sword role in human HCC cells, suggesting that novel H_2_S donors can be designed and applied for the treatment of cancer [52].

In this study, a new coupling compound HA-ADT was designed and synthesized as previous described [53]. HA-ADT could generate more H_2_S than both sodium hydrosulfide (NaHS, a fast H_2_S-releasing donor) and morpholin-4-ium (4-methoxyphenyl)-morpholin-4-ylsulfanylidenesulfido-*λ*5-phosphane (GYY4137, a slow H_2_S-releasing donor) [53]. The roles of HA-ADT in the proliferation, migration, and invasion of human HCC cells were investigated. Then we conducted *in vivo* experiments to further determine the effect of HA-ADT on the growth of human HCC xenografts.

## 2. Materials and Methods

### 2.1. Cell Culture

Human HCC cell lines SMMC-7721 and Huh-7 were obtained from Kebai Biological Technology Co., Ltd. (Nanjing, Jiangsu, China). SMMC-7721 cells were grown in RPMI1640 medium supplemented with 10% fetal bovine serum (FBS), penicillin (100 U/ml), and streptomycin (100 *μ*g/mL). Huh-7 cells were grown in DMEM supplemented with 10% FBS, penicillin (100 U/ml), and streptomycin (100 *μ*g/mL). Cells were cultured in an incubator at 37°C with 95% air and 5% CO_2_. Cells were treated with NaHS (200 *μ*M), GYY4137 (200 *μ*M), and HA-ADT (200 *μ*M) (provided by Prof. Shao-Feng Duan), respectively [53]. The control group was treated with phosphate-buffered saline (PBS). After treatment for 24 h, the cells were adopted for following experiments.

### 2.2. Cell Growth Assay

The Cell-Light 5-ethynyl-2-deoxyuridine (EdU) Apollo 567 Kits (RiboBio, Guangzhou, China) were used to detect cell proliferation. Cell proliferation rate = (EdU-positive cells)/(total cells) × 100% [54]. The 3-(4, 5-dimethyl-2-thiazolyl)-2, 5-diphenyl-2-H-tetrazolium bromide (MTT) (Sigma, St. Louis, MO, USA) and CCK-8 detection kits (Beyotime, Shanghai, China) were adopted to determine cell viability [55–57].

### 2.3. Colony Formation Assay

The cells (1 × 10^3^ per well) were cultivated in a culture medium for 2 weeks at 37°C. After washing with PBS, the colonies were fixed with methanol. Crystal violet was then added and incubated at room temperature for 30 min. The plates were washed, air-dried, and scanned. Then, the colony number was counted.

### 2.4. Wound Healing Assay

The cultured monolayer-confluent cells were scratched. The migration distance was photographed under an inverted microscope (Olympus CKX41, Tokyo, Japan). The cell migration rate (MR) was calculated as MR (%) = [(*A* − *B*)/*A*] × 100, where *A* and *B* are the widths at 0 h and 24 h, respectively [58].

### 2.5. Migration and Invasion Assays

The cells (1 × 10^5^) were seeded into the matrigel coated/uncoated upper chamber. The medium supplemented with 20% FBS was added into the lower chamber. After 24 h of treatment, the remaining cells on the upper side were scrubbed off, and the cells on the bottom side were fixed with 4% paraformaldehyde, and then stained with 0.1% crystal violet for 20 min. The cells were counted using an Axioskop 2 plus microscope (Zeiss, Thornwood, NY, USA).

### 2.6. Flow Cytometry Analysis

1 × 10^6^ cells were trypsinized and then fixed in ice-cold 75% ethanol overnight. After washing with PBS, the cells were incubated in propidium iodide (PI)/RNase A mixture at room temperature for 30 min. The cell cycle distribution was analyzed with a FACSVerse flow cytometer (CytoFLEX S, Beckmann, CA, USA). The apoptotic level was detected using the Annexin V and PI apoptosis kits (UE, Suzhou, Jiangsu, China) and analyzed using a FACSVerse flow cytometer.

### 2.7. TdT-Mediated dUTP-Biotin Nick End Labeling (TUNEL) Assay

TUNEL assay was carried out using the *in situ* cell death detection kits (Beyotime). The cells were examined with a fluorescence microscope (Nikon Eclipse Ti, Melville, NY, USA). The percentage of TUNEL positive cells was further quantified.

### 2.8. Western Blotting

Total protein was extracted from human HCC cells. Western blotting was used to determine the expression levels of relevant proteins. The primary antibodies include anti-Cyclin E1, anti-Cyclin D1, anti-cyclin-dependent kinase (CDK) 2, anti-CDK4, anti-p27, anti-p21, anti-AKT, anti-phospho (p)-AKT (Ser473), anti-glycogen synthase kinase-3 beta (Gsk‐3*β*), anti-p-Gsk-3*β* (Ser9), anti-*β*-catenin, anti-p-*β*-catenin (Ser552), anti-beclin-1, anti-p62, anti-LC3A/B, anti-Smad2, anti-p-Smad2 (Ser465/467), anti-Smad3, anti-p-Smad3 (Ser423/425), and anti-transforming growth factor-beta (TGF‐*β*) antibodies, as well as the horseradish peroxidase-conjugated secondary antibody obtained from Cell Signaling Technology (CST, Danvers, MA, USA). Anti-B-cell lymphoma-2 (Bcl-2), anti-B-cell lymphoma-extra large (Bcl-xl), anti-Bcl-2-associated X protein (Bax), anti-Bcl-xl/Bcl-2-associated death promoter (Bad), anti-cleaved caspase (cas)-3, anti-cleaved cas-9, anti-cleaved poly adenosine diphosphate‐ribose polymerase (PARP), and anti-*β*-actin antibodies were obtained from ProteinTech (Chicago, IL, USA). The protein bands were detected with the enhanced chemiluminescence system (Thermo, Rockford, IL, USA) and semiquantified by ImageJ software.

### 2.9. Animal Study

Animal experiments were approved by the Committee of Medical Ethics and Welfare for Experimental Animals of Henan University School of Medicine (HUSOM-2017-218). Animal study was carried out as previously described [53]. BALB/c nude mice (male, 4-week-old) were obtained from Vital River Laboratory Animal Technology Co., Ltd. (Beijing, China). 5 × 10^6^ SMMC-7721/Huh-7 cells in PBS (200 *μ*L) were injected subcutaneously into the right flank of each mouse. The mice were divided randomly into 4 groups (*n* = 6 per group). NaHS (200 *μ*M), GYY4137 (200 *μ*M), HA-ADT (200 *μ*M), and PBS were administered subcutaneously once-a-day for 21 days. During the animal experiment, the mice were daily weighed. The tumor volume was calculated as follows: Volume (*V*) = 1/2 × *W*^2^ × *L*, where *L* and *W* are the longest and widest dimension, respectively [59]. The tumor volume doubling time (TVDT) was calculated as follows: TVDT = (*T* − *T*_0_) × log2/log (*V*_2_/*V*_1_), where *V*_2_ and *V*_1_ are the tumor volume at two measurement times and (*T* − *T*_0_) is the time interval [60, 61]. At the end of the experiment, all mice were sacrificed. Then the tumors were excised, weighted, and photographed. Inhibition rate (IR) = [(*A* − *B*)/*A*] × 100%, where *A* and *B* are the average tumor weight of control group and the treatment group, respectively [53].

### 2.10. Hematoxylin and Eosin (HE) Staining

Tumor tissues were fixed in 10% neutral-buffered formalin, embedded in paraffin, sectioned at 5 *μ*m thickness, and then stained with HE. The tissues were observed using a Zeiss Axioskop 2 plus microscope.

### 2.11. Immunohistochemistry (IHC)

Microvessel density (MVD) has been widely used as an index for the angiogenic activity [62]. The proliferation index (PI) was calculated as the percentage of Ki67-positive cells to total cells [63]. Apoptotic index was determined as cleaved cas-3 -positive cells to total cells [64]. Tumor tissues were stained with anti-CD31 (CST), anti-Ki67 (CST), anti-p21, anti-cleaved cas-3, and anti-beclin-1 antibodies, respectively. The proliferation index (PI), apoptosis index, MVD, p21-positive cells, and autophagy index were counted according to the ratio of positive cells to total cells.

### 2.12. Statistical Analysis

All results are expressed as the mean ± standard error of the mean (SEM). Differences among groups were determined using one-way analysis of variance with SPSS 19.0 software, followed by Tukey's test. A *P* value of less than 0.05 was considered statistically significant.

## 3. Results

### 3.1. HA-ADT Inhibits the Growth, Migration, and Invasion of Human HCC Cells

**Figure 1:**
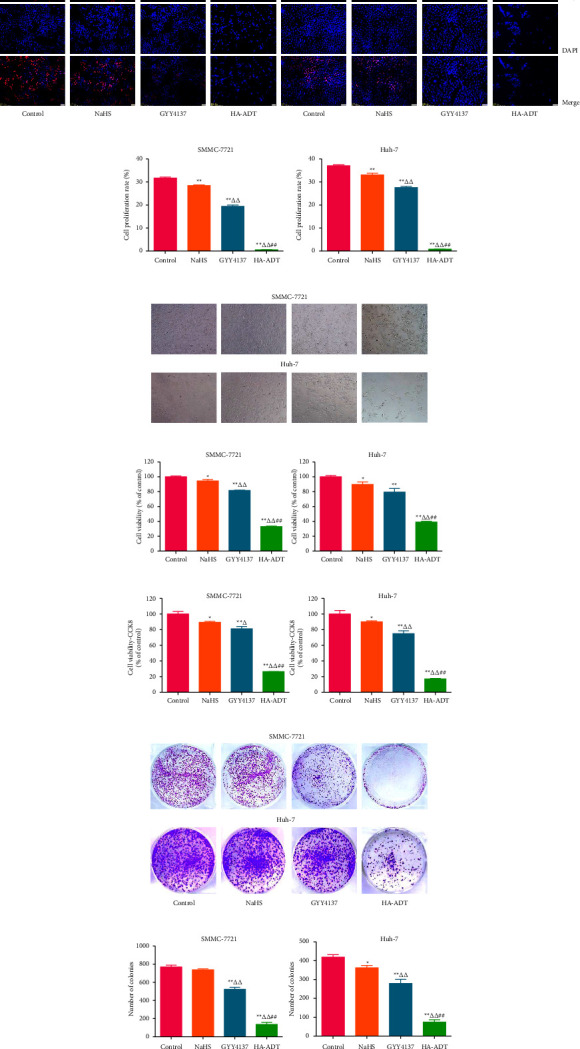
Effects of HA-ADT on proliferative ability and cell viability of human HCC cells. (a) EdU assay was adopted to determine DNA replication activity (original magnification, 100x). (b) Cell proliferation rate was calculated. (c) Phase contrast microscopy of Huh-7 and SMMC-7721 cells (original magnification, 100x). (d, e) Cell viability was measured by MTT and CCK8 assays. Cell viability in the control group was considered to be 100%. (f) The cells were treated for 2 weeks and then the clonogenic ability was detected. (g) The number of colonies was calculated. All data are shown as the mean ± SEM of three independent experiments; ^*∗*^*P* < 0.05, ^*∗∗*^*P* < 0.01 vs. control group; ^△^*P* < 0.05, ^△△^*P* < 0.01 vs. NaHS group; ^##^*P* < 0.01 vs. GYY4137 group.

**Figure 2:**
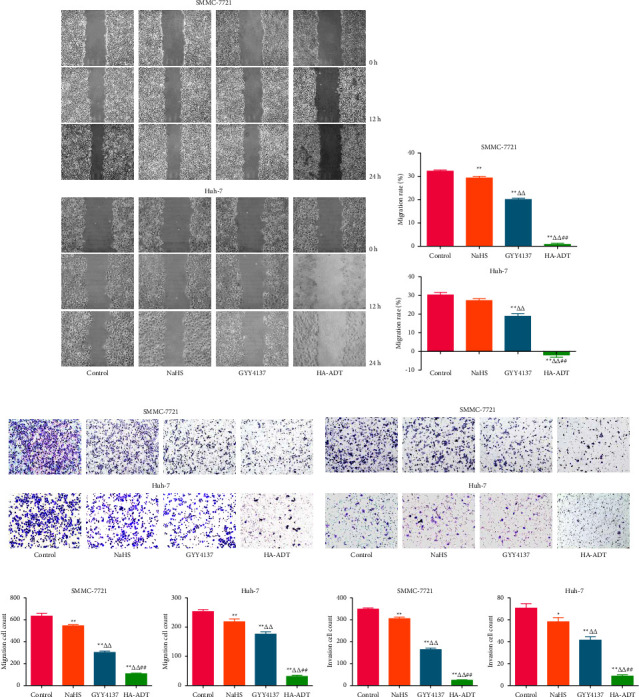
Effects of HA-ADT on the migrative and invasive capacities of human HCC cells. (a) Wound-healing assay was performed to determine cell migration (original magnification, 100x). (b) The migration rate was determined. (c, d) Transwell assay was carried out to evaluate the migrative and invasive abilities of human HCC cells (original magnification, 200x). (e, f) The numbers of the migrative and invasive cells were determined. All data are shown as the mean ± SEM of three independent experiments; ^*∗*^*P* < 0.05, ^*∗∗*^*P* < 0.01 vs. control group; ^△△^*P* < 0.01 vs. NaHS group; ^##^*P* < 0.01 vs. GYY4137 group.

Compared with the control, NaHS, and GYY4137 group, HA-ADT significantly suppressed the viability and proliferation of Huh-7 and SMMC-7721 cells (Figures 1(a)–1(e)). HA-ADT also more effectively reduced the colony formation of SMMC-7721 and Huh-7 cells (Figures 1(f) and 1(g)). In addition, HA-ADT showed more inhibitory effects on the migration and invasion of Huh-7 and SMMC-7721 cells than the control, NaHS, and GYY4137 group (Figure 2). In summary, the results show that HA-ADT could inhibit the growth, migration, and invasion of HCC cells.

### 3.2. HA-ADT Blocks Cell Cycle of Human HCC Cells

**Figure 3:**
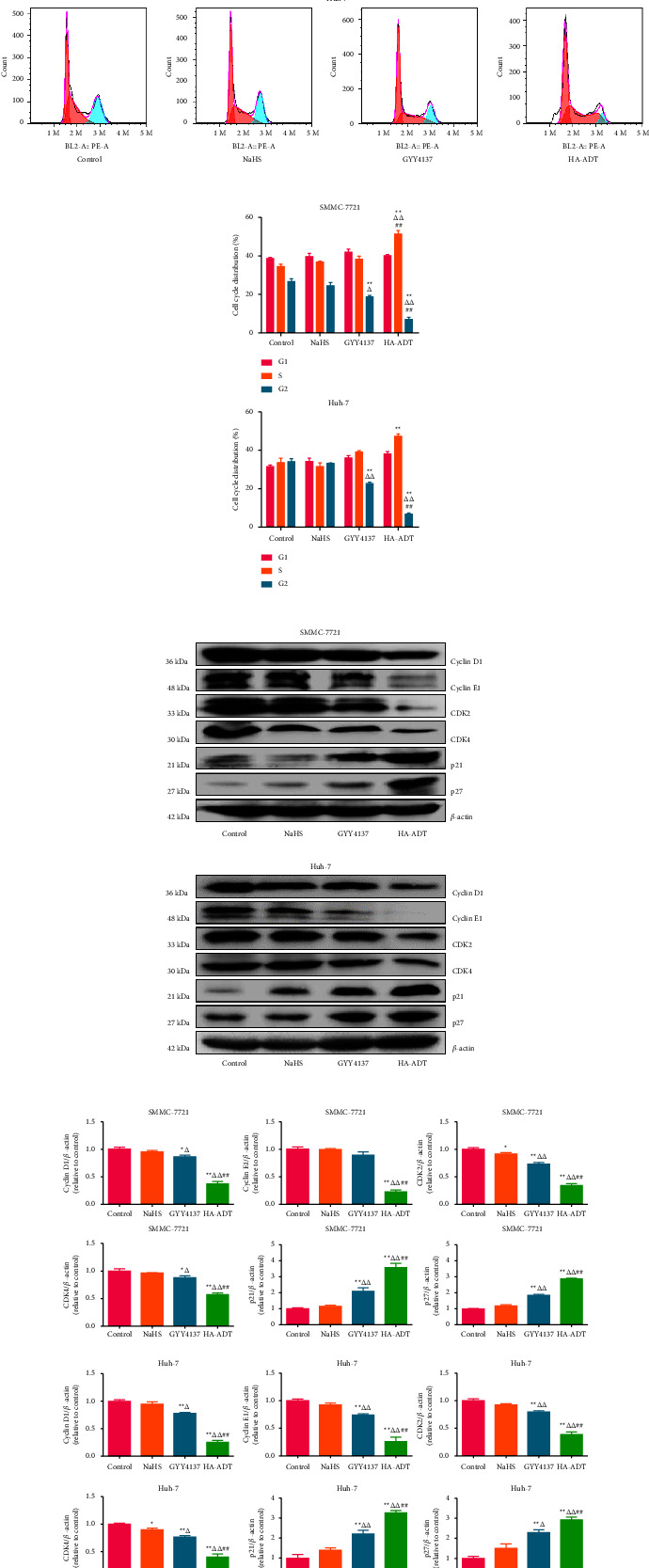
Effects of HA-ADT on the cell cycle of human HCC cells. (a) Cell cycle distribution was detected by flow cytometry. (b) The results of cell cycle distribution were analyzed. (c) The expression levels of cyclin D1/E1, CDK2/4, p21, and p27 were detected. *β*-actin was adopted as the internal control. (d) The band density was analyzed. All data are shown as the mean ± SEM of three independent experiments; ^*∗*^*P* < 0.05, ^*∗∗*^*P* < 0.01 vs. control group; ^△^*P* < 0.05, ^△△^*P* < 0.01 vs. NaHS group; ^##^*P* < 0.01 vs. GYY4137 group.

As shown in Figures 3(a) and 3(b), HA-ADT significantly upregulated the percentage of cells in S phase but downregulated the percentage of cells in G2 phase, suggesting that HA-ADT blocked the cell cycle in S phase. Western blot was further conducted to detect the protein levels of cyclin D1/E1, CDK2/4, p21, and p27. The results indicated that HA-ADT decreased the levels of cyclin D1/E1 and CDK2/4, but increased the levels of both p21 and p27 (Figures 3(c) and 3(d)). These results indicate that HA-ADT can block the cell cycle of human HCC cells at S phase by regulating the expression levels of cell cycle-related proteins.

### 3.3. HA-ADT Promotes Apoptosis by Suppressing the AKT/GSK-3*β*/*β*-Catenin Signaling Pathway in Human HCC Cells

**Figure 4:**
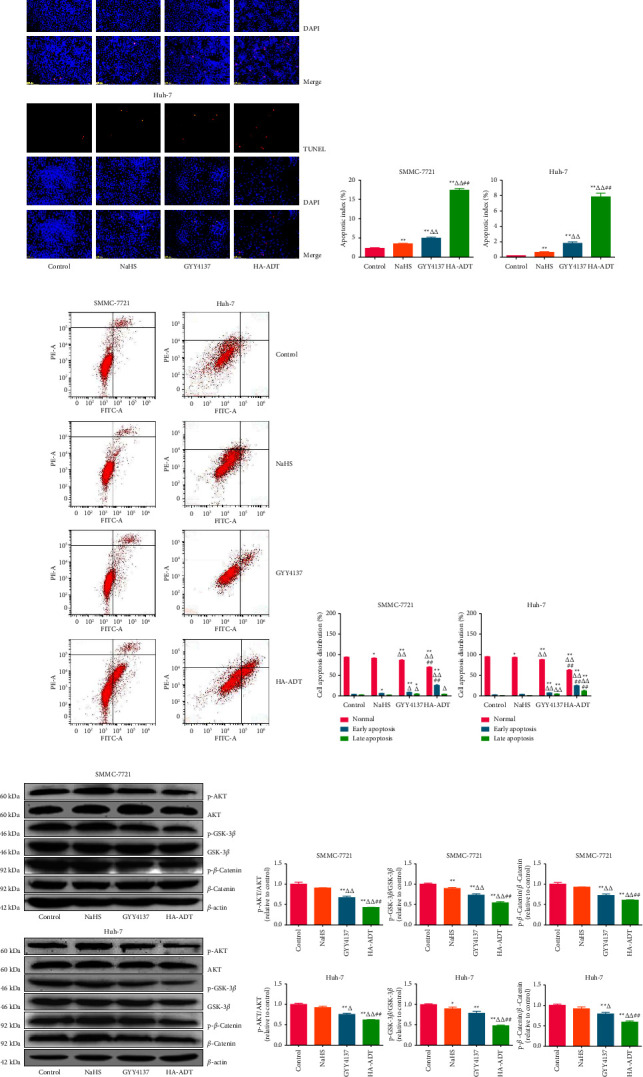
Effects of HA-ADT on the apoptotic level and AKT/GSK-3*β*/*β*-catenin pathway in human HCC cells. (a) TUNEL staining was used to detect the apoptotic level (original magnification, 100x). (b) The apoptotic index was counted as the ratio of TUNEL positive cells to total cells. (c) Flow cytometry assay was adopted to detect apoptosis. (d) Cell apoptosis distribution was analyzed. (e) Western blotting was used to determine the protein levels of AKT, p-AKT, GSK-3*β*, p-GSK-3*β*, *β*-catenin, and p-*β*-catenin. *β*-actin was adopted as the internal control. (f) The density was analyzed. All data are shown as the mean ± SEM of three independent experiments; ^*∗*^*P* < 0.05, ^*∗∗*^*P* < 0.01 vs. control group; ^△^*P* < 0.05, ^△△^*P* < 0.01 vs. NaHS group; ^##^*P* < 0.01 vs. GYY4137 group.

Apoptosis was detected by TUNEL and flow cytometry assays. As shown in Figures 4(a)–4(d), the apoptotic level in the HA-ADT group was significantly increased when compared with the control, NaHS, and GYY4137 group. The ratios of Bax/Bcl-2 and Bad/Bcl-xl are important indicators of apoptosis. Increased Bax/Bcl-2 and Bad/Bcl-xl ratios have been found in mitochondria-mediated apoptosis [65, 66]. As shown in Figure S1, Bax/Bcl-2 and Bad/Bcl-xl ratios in the HA-ADT group were obviously increased. In addition, the expression levels of cleaved cas-3, 9, and cleaved PARP exhibited similar trends. The AKT/GSK-3*β*/*β*-catenin cascade is a key pathway involved in the survival, growth, and metabolic stability of tumor cells [67, 68]. As shown in Figures 4(e) and 4(f), the phosphorylation levels of AKT, GSK-3*β*, and *β*-catenin were downregulated in the HA-ADT group. The data suggest that HA-ADT induces apoptosis via suppressing the AKT/GSK-3*β*/*β*-catenin pathway in human HCC cells.

### 3.4. HA-ADT Decreases Autophagy of Human HCC Cells by Inhibiting the TGF-*β*/Smad2/3 Signaling Pathway

**Figure 5:**
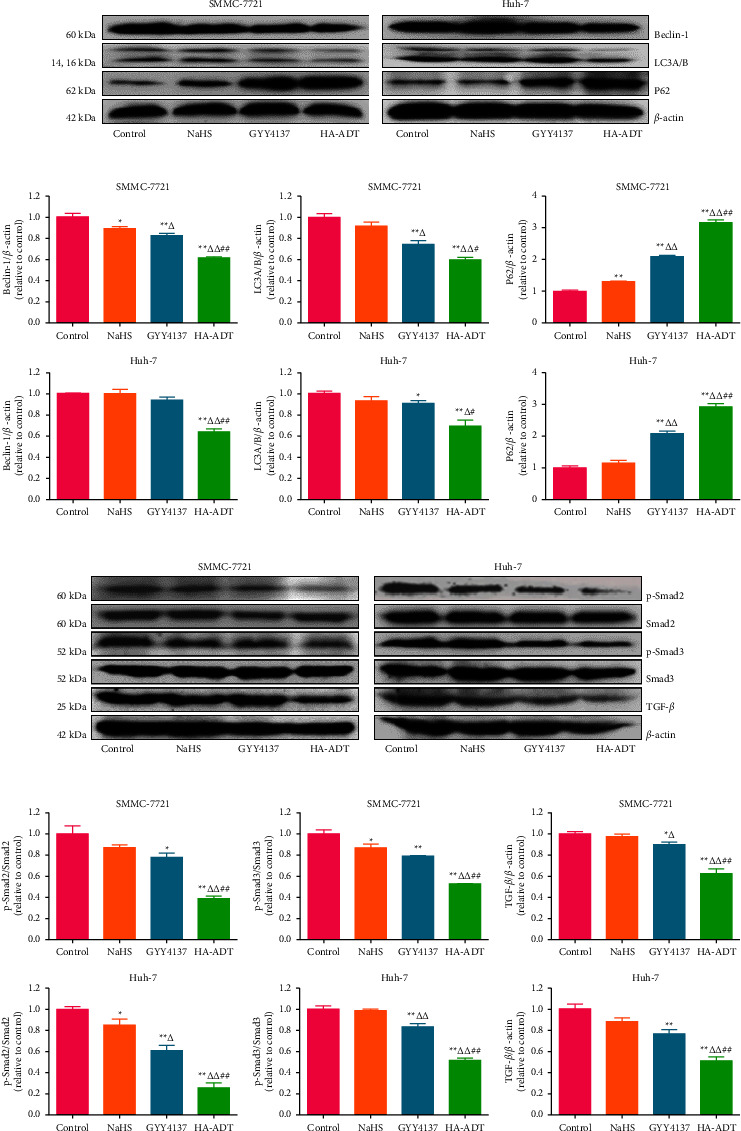
Effects of HA-ADT on the autophagic level and TGF-*β*/Smad2/3 signaling pathway in human HCC cells. (a) The protein levels of beclin-1, LC3A/B, and p62 were detected by western blotting. *β*-actin was adopted as the internal control. (b) The band density was analyzed. (c) The protein levels of p-Smad2, Smad2, p-Smad3, Smad3, and TGF-*β* were detected by western blotting. *β*-actin was adopted as the internal control. (d) The band density was analyzed. All data are shown as the mean ± SEM of three independent experiments; ^*∗*^*P* < 0.05, ^*∗∗*^*P* < 0.01 vs. control group; ^△^*P* < 0.05, ^△△^*P* < 0.01 vs. NaHS group; ^#^*P* < 0.05, ^##^*P* < 0.01 vs. GYY4137 group.

The role of autophagy in the development of cancer is extremely complex [69, 70]. Autophagy is a conservative catabolic process, which plays a dual role in regulating cell growth [71, 72]. Autophagy is an important mechanism by which cellular material is transfered to lysosome for degradation, thus providing energy and allowing the transformation of cellular components [70, 73, 74]. Beclin-1, LC3, and p62 are considered as specific autophagic markers [74]. As shown in Figures 5(a) and 5(b), the expressions of LC3 and beclin-1 in HA-ADT group were lower than those in the control, NaHS, and GYY4137 group, while the expression level of p62 exhibited the opposite trend. TGF-*β* plays an important role in cell homeostasis, fibrosis, angiogenesis, carcinogenesis, and differentiation. TGF-*β* can reduce apoptosis by inducing autophagy [75]. It has been shown that Smad2/3 is also involved in autophagy [76]. As shown in Figures 5(c) and 5(d), the expression levels of p-Smad2/3 and TGF-*β* in the HA-ADT group were lower than those in the control, NaHS, and GYY4137 group. These results indicate that HA-ADT can inhibit autophagy in human HCC cells via the TGF-*β*/Smad2/3 pathway.

### 3.5. HA-ADT Suppresses the Growth of Human HCC Xenografted Tumors

**Figure 6:**
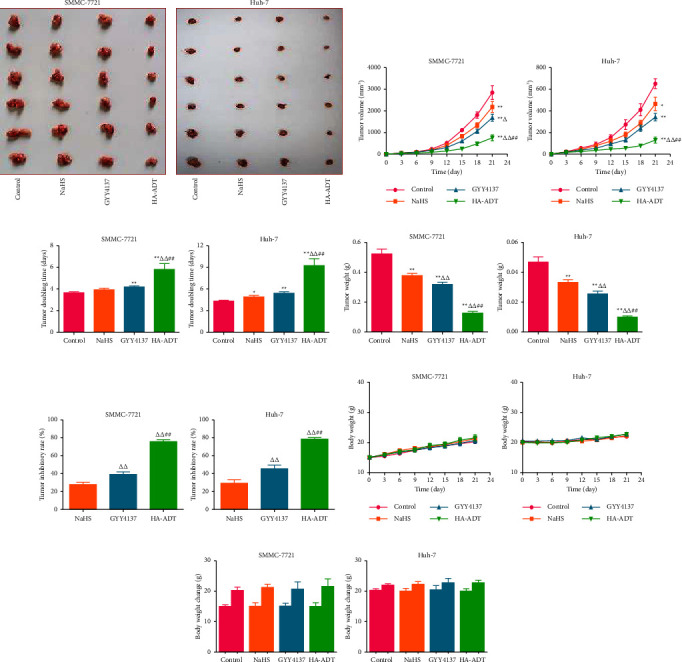
Effects of HA-ADT on human HCC xenograft tumor growth. (a) Representative xenograft tumors of each group were shown. (b, c) The tumor volume and TVDT were determined. (d, e) The tumor weight and tumor inhibitory rate were measured. (f, g) The body weight change curve and the body weights of mice (day 0 and day 21) were calculated. All data are presented as the mean ± SEM (*n* = 6); ^*∗*^*P* < 0.05, ^*∗∗*^*P* < 0.01 vs. control group; ^△^*P* < 0.05, ^△△^*P* < 0.01 vs. NaHS group; ^##^*P* < 0.01 vs. GYY4137 group.

**Figure 7:**
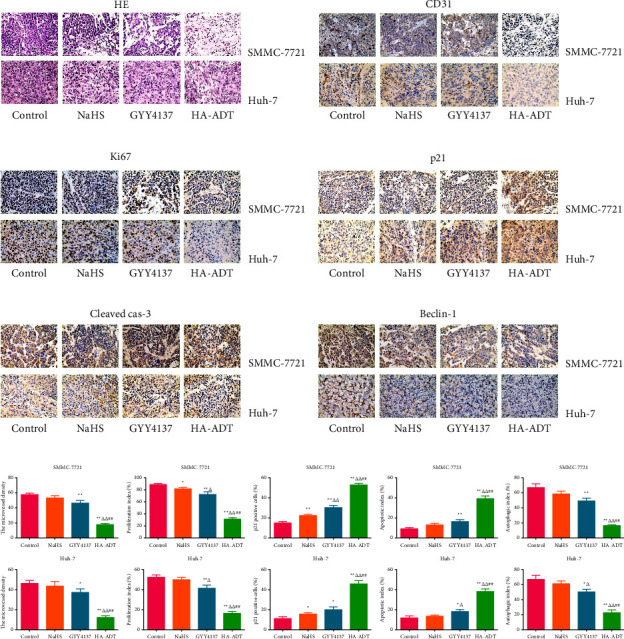
Effects of HA-ADT on MVD, PI, cell cycle, apoptosis, and autophagy of human HCC xenograft tumors. (a–f) Representative photographs of HE staining, and IHC staining with anti-CD31, anti-Ki67, anti-p21, anti-cleaved cas-3, and anti-beclin-1 antibodies in human HCC xenograft tumors (original magnification, 400x). (g) The MVD, proliferation index, p21 positive cells, apoptotic index, and autophagic index were determined. All data are presented as the mean ± SEM (*n* = 6); ^*∗*^*P* < 0.05, ^*∗∗*^*P* < 0.01 vs. control group; ^△^*P* < 0.05, ^△△^*P* < 0.01 vs. NaHS group; ^##^*P* < 0.01 vs. GYY4137 group.

SMMC-7721 and Huh-7 HCC cells have been successfully used to establish the subcutaneous xenograft models [77, 78]. Compared with the control, NaHS, and GYY4137 group, HA-ADT dramatically suppressed the growth of xenografted tumors (Figures 6(a)–6(e)). In addition, there was no significant difference in body weight between each group (Figures 6(f) and 6(g)). The expression levels of CD31, Ki67, p21, cleaved cas-3, and beclin-1 were further detected by IHC. As shown in Figure 7, the expression levels of CD31, Ki67, and beclin-1 in HA-ADT group were decreased, while p21 and cleaved cas-3 levels were increased in the HA-ADT group. These data demonstrate that HA-ADT can effectively suppress human HCC xenograft tumor growth by promoting apoptosis and reducing autophagy.

## 4. Discussion

At present, H_2_S is considered the third gaseous transmitter after CO and NO. H_2_S is involved in many physiological and pathological processes in the human body [21–23, 79]. HA and its derivatives have the characteristics of plasticity, nonimmunogenicity, and good biocompatibility, which are widely adopted in the biomedical field, such as tissue engineering and drug delivery [39–41]. ADT is a methyl derivative of ADT-OH, which is a common H_2_S donor [42, 79]. In the present study, HA-ADT was synthesized by chemical reaction as previously described [53].

Liver cancer is one of the leading causes of cancer death in the world [1–4]. Liver cancer is difficult to diagnose in the early stage, which will result in the death of patients. In addition, there are few effective treatments for patients with advanced liver cancer [3, 6, 13]. Thus, it is urgent to explore novel drugs to prevent and treat liver cancer. It has been revealed that 25–100 *μ*M·NaHS promotes the growth of HCC cells and blood vessel formation, while 800–1000 *μ*M·NaHS can inhibit angiogenesis and HCC growth [52]. Another study suggests that GYY4137 exhibits potent anti-HCC activity by blocking the signal transducer and activator of transcription 3 pathway [80]. In this study, we examined the roles of HA-ADT in the growth, migration, invasion, and cell cycle of human HCC cells. The results showed that HA-ADT was more effective than both NaHS and GYY4137 in inhibiting the survival, proliferation, migration, invasion, and cell cycle progression of human HCC cells. These results suggest that HA-ADT plays an effective role in inhibiting the development and progression of human HCC cells.

Apoptosis, a form of programmed cell death, is evolutionarily conserved and plays a key role in the homeostasis and development of mammalian tissues [81]. Apoptotic pathways can be divided into two categories: mitochondria-mediated intrinsic pathway and death receptor-mediated extrinsic pathway [82]. Bcl-2 family proteins are pivotal members in the process of apoptosis [53]. Caspases play key roles in apoptotic signaling pathways [83]. Caspases can be activated by many apoptotic stimuli and PARP is cleaved by cleaved caspase-3, resulting in the occurrence of apoptosis [53, 84]. It has been shown that NaHS effectively decreases the growth of C6 glioma cells by inducing caspase-dependent apoptosis [85]. Furthermore, GYY4137 could induce apoptosis in HCC cells by increasing the levels of cleaved cas-9, cas-3 and PARP cleavage [80]. Similarly, our results indicated that HA-ADT can promote apoptosis in HCC cells by up-regulating the levels of cleaved cas-3, 9, and cleaved PARP, indicating the activation of mitochondrial apoptosis. The AKT/GSK-3*β*/*β*-catenin pathway is involved in a number of hallmarks of cancer, such as tumor grade and lympho-node metastasis [67, 68, 86]. It has been reported that the AKT pathway is involved in HCC growth and metastasis [68]. In addition, GSK-3*β* plays a key role in the phosphorylation/degradation of *β*-catenin in the AKT/GSK-3*β*/*β*-catenin pathway [68]. The results suggested that HA-ADT could reduce the expressions of p-AKT, p-GSK-3*β*, and p-*β*-catenin in human liver cancer cells. The current research suggests that HA-ADT can inhibit the growth of human HCC cells by inducing apoptosis via inhibition of the AKT/GSK-3*β*/*β*-catenin signaling pathway.

Autophagy could be neutral, tumor-promoting, or tumor-suppressive in different contexts in cancer cells [87]. A recent study has shown that diallyl trisulfide, a characterized H_2_S donor, inhibits the proliferation of urothelial carcinoma cells by promoting apoptosis and inducing autophagy [88]. Our previous study has demonstrated that HA-ADT could suppress the progression of esophageal squamous cell carcinoma via apoptosis promotion and autophagy inhibition [89]. Similarly, in the present study, our data showed that HA-ADT significantly reduced the autophagic level when compared to the control, NaHS, and GYY4137 group. It has been reported that the TGF-*β* pathway can activate autophagy in many human cancer cells, suggesting that induction of autophagy is a novel biological function of TGF-*β* [90]. Furthermore, as canonical effectors of TGF-*β* signaling, Smad2/3 are involved in the process of autophagy [91]. Moreover, another study suggests that specificity protein 1-mediated serine/threonine kinase 39 upregulation promotes the proliferation, migration, and invasion of HCC cells by activating the TGF-*β*1/Smad2/3 pathway [47]. Our data indicated that HA-ADT decreased the expressions of p-Smad2/3 and TGF-*β* compared with the control, NaHS, and GYY4137 group. These findings indicate that HA-ADT can inhibit autophagy in human HCC cells through the TGF-*β*/Smad2/3 signaling pathway.

Recent studies suggest that SMMC-7721 and Huh-7 cells have been widely adopted to establish the xenograft tumor models [77, 78]. Therefore, we studied the role of HA-ADT in the growth of HCC xenograft tumor. We observed that HA-ADT exerted more inhibitory effects on HCC xenograft tumor growth than the control, NaHS, and GYY4137 group. Furthermore, there was no obvious change in the body weight in each group. Similar to the *in vitro* findings, our data suggested that the expressions of Ki67, CD31, and beclin-1 were decreased in HA-ADT group. The levels of p21 and cleaved cas-3 in the HA-ADT group were significantly higher than those in the control, NaHS, and GYY4137 group. The data together indicate that HA-ADT can effectively inhibit the growth of human HCC xenograft tumors.

In sum, HA-ADT can suppress the proliferation, migration and invasion of human HCC cells via inhibition of the AKT/GSK-3*β*/*β*-catenin and TGF-*β*/Smad2/3 signaling pathways. HA-ADT might be considered as a promising anticancer candidate for the treatment of HCC.

## Data Availability

All data generated or analyzed in this study are included in this article.

## Supplementary Materials

Supplementary Materials Figure S1: Effects of HA-ADT on the expression levels of mitochondrial apoptosis-related proteins in human HCC cells. (a) Western blotting was used to detect the expressions of Bcl-2, Bax, Bcl-xl, Bad, cleaved cas-3, 9, and cleaved PARP in SMMC-7721 and Huh-7 cells. *β*-actin was adopted as the internal control. (b) The band density was analyzed. The Bax/Bcl-2 and Bad/Bcl-xl ratios were calculated. All data are shown as the mean ± SEM of three independent experiments; ^*∗*^*P* < 0.05, ^*∗∗*^*P* < 0.01 vs. control group; ^△^*P* < 0.05, ^△△^*P* < 0.01 vs. NaHS group; ^##^*P* < 0.01 vs. GYY4137 group.
