# Supplementary figures and images for: A Water-Soluble Hydrogen Sulfide Donor Suppresses the Growth of Hepatocellular Carcinoma via Inhibiting the AKT/GSK-3β/β-Catenin and TGF-β/Smad2/3 Signaling Pathways
Source: J Oncol. 2023 Mar 7;2023:8456852. doi: 10.1155/2023/8456852 (PMC10014162; doi:10.1155/2023/8456852)

Figure S1

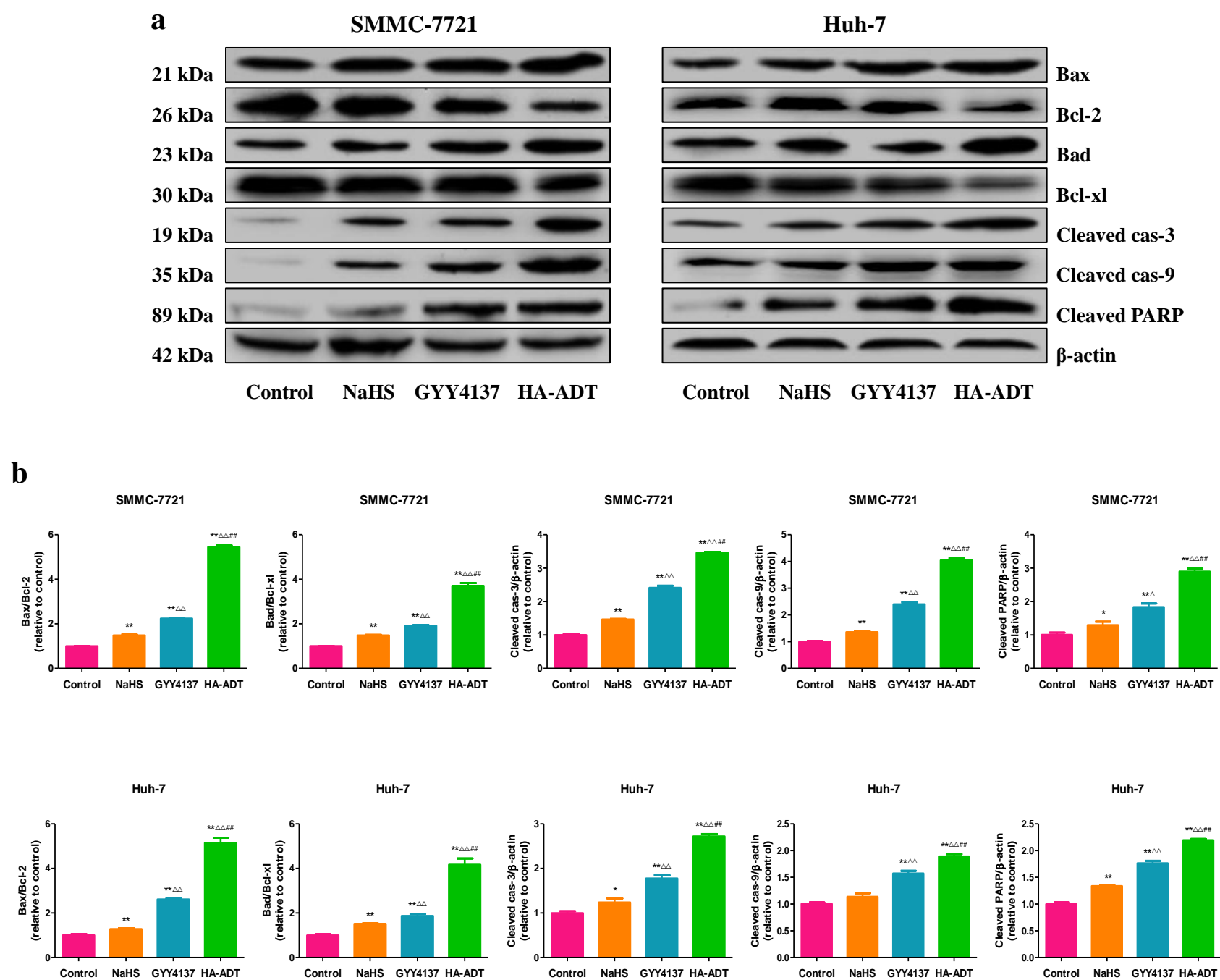

Supplement: Supplementary Materials — Figure S1: Effects of HA-ADT on the expression levels of mitochondrial apoptosis-related proteins in human HCC cells. (a) Western blotting was used to detect the expressions of Bcl-2, Bax, Bcl-xl, Bad, cleaved cas-3, 9, and cleaved PARP in SMMC-7721 and Huh-7 cells. β-actin was adopted as the internal control. (b) The band density was analyzed. The Bax/Bcl-2 and Bad/Bcl-xl ratios were calculated. All data are shown as the mean ± SEM of three independent experiments; ∗P < 0.05, ∗∗P < 0.01 vs. control group; △P < 0.05, △△P < 0.01 vs. NaHS group; ##P < 0.01 vs. GYY4137 group. [file 8456852.f1.pdf]
